# Isolated pancreatic injury in an adolescent treated with Roux-en-Y pancreatojejunostomy: a case report and review of the literature

**DOI:** 10.1186/s13256-021-03042-7

**Published:** 2021-09-16

**Authors:** Mohamed Eltayeb Abdelrahman Naiem, Nassir Alhaboob Arabi

**Affiliations:** 1 Faculty of Medicine, University of Khartoum, Khartoum, Sudan; 2 Department of Gastrointestinal and Hepato-Pancreatic-Biliary Surgery, Ibn Sina Specialized Hospital, Khartoum, Sudan

**Keywords:** Pancreatic blunt trauma, Major duct injury, Roux-en-Y pancreatojejunostomy

## Abstract

**Background:**

Pancreatic injury presented as isolated injury in the pediatric population is exceptionally rare, with a conveyed incidence of less than 2% of all abdominal trauma injuries cases and a very controversial management approach for grade III injuries.

**Case presentation:**

A 16-year-old adolescent Sudanese boy was referred to our emergency department with a 5-day history of upper and left hypochondrial pain after blunt abdominal trauma to the epigastric region with a solid object. Grade III pancreatic body trauma with major duct involvement can be successfully treated operatively. The boy was discharged home on day 10 with regular oral intake and diet. A follow-up for 6 months continued by phone, and it was uneventful with no further complications.

**Conclusions:**

Roux-en-Y pancreatojejunostomy reconstruction can be a safe and valuable surgical option when the surgical approach is considered for grade III pancreatic injury.

## Introduction

Blunt abdominal injury causing significant and major pancreatic injury is rare in adolescents and young adults, with a controversial approach to its management. The position of the pancreas in the retroperitoneal region makes isolated injury a rare entity and resembles a challenge for the surgeon. It is graded into five grades ranging from minor contusion or small laceration without duct injury to major pancreatic duct or head disruption and transection according to the Association for the Surgery of Trauma organ injury scale (AAST-OIS). Management options range from a conservative approach to major pancreatic resection and complex reconstruction.

## Case presentation

**Fig. 1:**
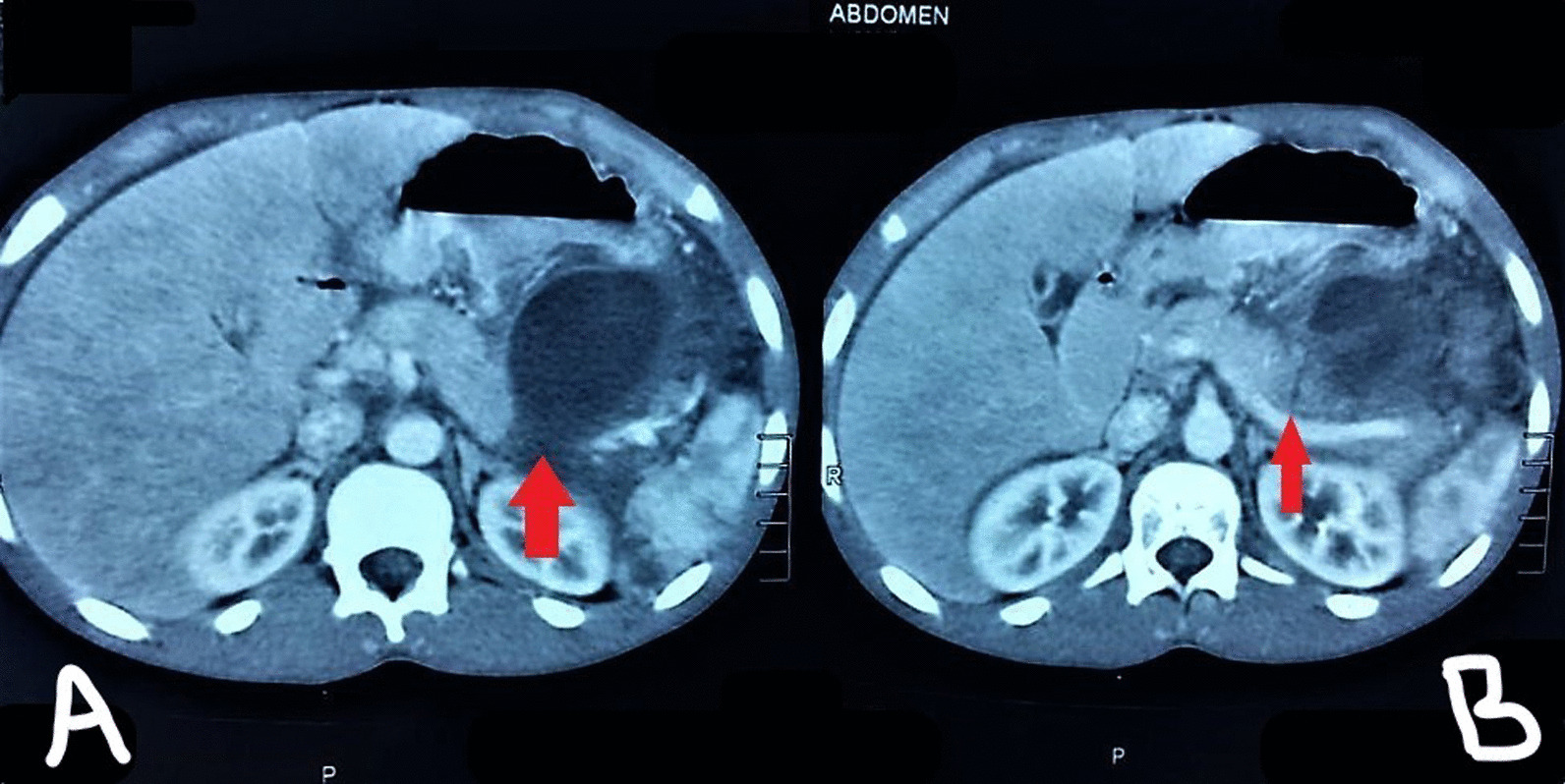
Computed tomographic scan of the abdomen showing the pancreatic injury and peripancreatic collection. **A** - Left: Arrow represents peripancreatic collection communicating with injured pancreatic parenchyma and duct. **B** - Right: Arrow represents Pancreatic duct injury at the body of the pancreas. All patient related identifications were anonymized

A 16-year-old adolescent Sudanese boy was referred to our emergency department with a 5-day history of upper and left hypochondrial pain after blunt abdominal trauma to the epigastric region with a solid object. His pain was dull-aching in nature, localized to the epigastrium and left hypochondrial areas, aggravated and increased by movement and partially relieved by analgesia, but he had no fever, radiation, nausea, vomiting, or other associated symptoms. He had a clear medical and surgical background with a complete vaccination history. He is not allergic to any drugs or chronic medications. On examination, he was fully conscious, oriented, and aware of his surroundings, slightly pale but not jaundiced. His pulse rate was 120 beats per minute, and he was hypotensive with a blood pressure of 95/50 mmHg and slightly dehydrated. Abdominal examination revealed moderated epigastric and left hypochondrial tenderness with guarding but no rigidity, with hypoactive sluggish bowel sounds. No organomegaly masses were detected. The systemic review was clear, and no abnormality was detected. Blood investigations were requested, and hemoglobin (Hb) of 8.5 g/dl was revealed with normal white blood cell (WBC) count and platelets (PLTs). Urinalysis and blood electrolytes were normal. A CECT abdomen was performed before referral, and it showed lesser sac collection/hematoma with suspected grade III splenic injury and suspected pancreatic parenchymal injury; no other organ injury was detected (Fig. 1). The absence of magnetic resonance cholangiopancreatography (MRCP)/endoscopic retrograde cholangiopancreatography (ERCP) facilities supported the decision for emergency laparotomy after adequate resuscitation as the patient’s pain scale increased since the injury to presentation and vital sign charts were suggestive of intraperitoneal bleed correlating with the provisional diagnosis of splenic injury grade III.

An exploratory laparotomy through upper midline incision revealed a normal spleen with a clear, thick fluid collection in the lesser sac and contused pancreas with peripancreatic hematoma and anterosuperior distal pancreatic body laceration with major duct injury of (1 × 1.2 cm punched-out pancreatic parenchymal tissue) approximately, involving pancreatic parenchyma and duct with preservation of the posterior duct wall and communication with the lesser sac collection and intact posterior parenchyma and pancreatic magna and splenic arteries confirming the diagnosis of grade III injury American Association for the Surgery of Trauma organ injury scale (AAST-OIS) intraoperatively.

Lesser sac was accessed through the opening of the lesser omentum, careful examination of the pancreas, spleen, and major vascular structures. Peripancreatic wash, sample for amylase taken, debridement of the injury, and intraoperative discussion were made to drain the bed versus performing Roux-en-Y pancreatojejunostomy, and surgical reconstruction was the agreed option.

**Fig. 2:**
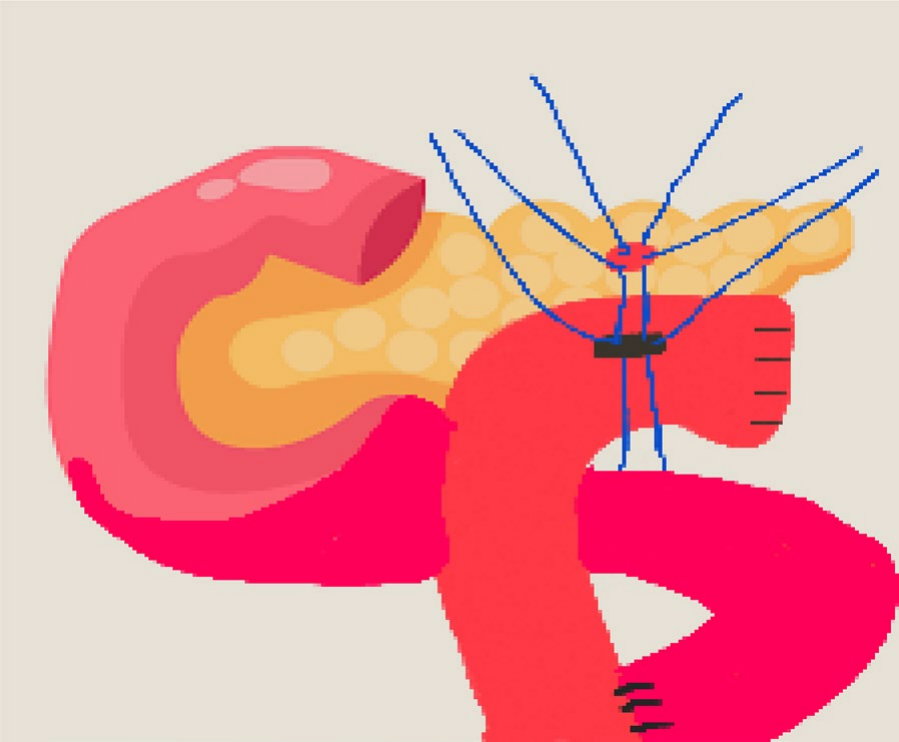
Roux-en-Y pancreatojejunostomy diagram demonstration

Roux-en-Y pancreatojejunostomy reconstruction was made in a retro-colic position, a Roux limb of 50 cm length and 8 mm enterotomy in the antimesenteric border, side to side, single layer with a full-thickness pancreatic–jejunal (duct to mucosa) anastomosis using 4/0 polydioxanone (PDS) interrupted stitches between the jejunum and pancreatic duct and parenchyma with augmenting corner stitches. Jejunojejunostomy was reconstructed with a 40 cm jejunal limb from the ligament of Treitz applying a hand-sewn, two-layer technique with 3/0 vicryl sutures (Fig. 2), pancreatic bed and peritoneal drainage *in situ*, and standard abdominal mass closure. Peritoneal fluid amylase tested positive, and oral intake started on day 3 postoperation. Drainage became dry on day 6 postoperation, and the patient was discharged home on day 10 with regular oral intake and diet.

A follow-up for 6 months continued by phone, and it was uneventful; he went back to his work as a shepherd after 3 months and gained significant weight.

## Discussion

Pancreatic injury presented as isolated injury in the pediatric population is infrequent, with a conveyed incidence of less than 2% of all abdominal injuries cases [1–3]. Nevertheless, it can be associated with significant comorbidities and mortality in a low-resource country.

It requires accurate observation and diagnosis as it mandates surgical intervention, especially when major duct injury is concerned [4]. Such uncommon injuries, especially those that are low-grade in nature, subclinical, or presenting with delayed complications, can be managed conservatively with favorable outcomes, as experienced in a reported series with male predominance related to male involvement in more traumatic activities. A multicenter study reported by Iqbal *et al*. concluded that grade II injuries should be managed nonoperatively, whereas grade III injuries should be managed by operative resection. However, grade III trauma with major duct involvement can be challenging and controversial [2, 5–7]. According to Krige *et al*., the postoperative complication and fatality rate in a single institute of 49 patients with isolated pancreatic injuries was 55% and 4%, consistently [8]. In a Korean study, Chi *et al*. described that the morbidity and mortality of 20 isolated pancreatic injuries were 65% and 0%, respectively [9]. The majority of type II and III injuries are now managed conservatively in the absence of indications for emergency exploration. Conservative management approach includes retaining the patient Nil Per Os (NPO), administration of intravenous solutions and somatostatin analog drugs and proton-pump inhibitors (PPIs), and relentless monitoring of vital signs, warning signs, and symptoms of peritonitis until the patient can safely tolerate oral intake [10].

Anterior Roux-en-Y pancreatojejunostomy was convenient in our case report after debridement of the wound area and the visually intact posterior pancreatic parenchyma [7]. The pancreatic duct injury in which pancreatic tissue was not transacted was distal to the head and neck (grade III). Utilizing this operative technique, we protected the patient from major pancreatic resection operations complications and prolonged general anesthesia complications in a critically ill trauma patient. In this operation, there was no necrotic tissue on the posterior wall of the pancreas, so the anastomosis was smaller than dunking pancreatic anastomosis, which was employed in Whipple’s procedure. Furthermore, the risk of anastomotic leakage is decreased. Additionally, insufficient debridement increases the probability of anastomosis leakage and can be reduced by undertaking adequate debridement [7].

An international agreement indicated that grade I–II injuries should be managed nonoperatively. In a multicenter cohort study of children with grade II and grade III pancreatic injuries, operative and conservative strategies emerged to be equivalent [11, 12]. Both grades of injury (II and III) represent an area of controversy and discussion to compare nonoperative versus operative management protocol and indications as many factors guide patient management according to patient status and centers’ capability to provide required imaging and interventional modalities if needed. A universal standardized protocol and consensus must be established to manage these serious forms of injuries [11, 13, 14].

## Conclusion

Grade III pancreatic isolated injury management remains controversial and situation-dependent, but due to the patient condition and lack of obtainability of ERCP at the time of presentation, operative management and Roux-en-Y pancreatojejunostomy reconstruction was the optimal and safe approach.

## Data Availability

The datasets used during the current study are available from the corresponding author on reasonable request. All medical data, supporting materials, and images are available upon request.

## Abbreviations

CECT Abdomen: Contrast-enhanced computed tomography scan
ERCP: Endoscopic retrograde cholangiopancreatography
AAST-OIS: American Association for the Surgery of Trauma organ injury scale
